# Experiments with the Sulphite of Lime as an Antiseptic

**Published:** 1863-10

**Authors:** E. Andrews

**Affiliations:** Professor of Surgery in Chicago Medical College


### ARTICLE XXIX.

### EXPERIMENTS WITH SULPHITE OF LIME AS AN ANTISEPTIC.

By E. ANDREWS, M.D., Professor of Surgery in Chicago Medical College.

M. Polli, of Milan, Italy, made a large number of experiments upon dogs, tending to show great antiseptic powers in the sulphites. Analogous to this quality is their well-known power of arresting fermentation in cider and other liquors. M. Polli, in one of his experiments, took two dogs, to one of whom he gave a quantity of the sulphites and to the other none. After some hours, both were killed and laid aside. He reports that the carcase of the dog which took nothing readily putrified, while that of the one which took the sulphite remained a long time perfectly fresh. During the hot weather of August, it occurred to me to try the experiment of the direct application of the sulphites to animal matters, thinking that if successful in preserving them from putrifaction, they would be an excellent application to suppurating wounds, and would prevent the formation of the septic poison of erysipelas.

I accordingly took four ounces of fresh beef, and cut it into two equal parts. Each fragment was placed in a wide vial, containing water enough to cover the beef. Into one of them

I dropped a drachm of sulphite of lime and shook up the bottle. The other contained only the beef and water. The two were placed on a shelf and left open. At the end of forty-eight hours, both were putrid and very offensive; and, so far as I could determine, there was no ground of choice between the odors of the two vials. This would seem to prove that sulphite of lime has no power of preserving animal substances, when applied to them directly. It, probably, requires the presence of an acid to combine with the lime and liberate free sulphurous acid to make it active. The acids of the stomach might serve this purpose, when given internally.

My object in selecting the sulphite of lime in preference to that of soda was, because the former, being insoluble, could be applied to a wound or ulcer without producing smarting. Notwithstanding the failure of the meat experiment, I tried the article externally in two cases where I had operated for the removal of necrosed bone. I mixed the sulphite of lime with clear water, and injected it freely into the wounds several times a-day for about ten days. The cases both did extremely well; but, as I took great care of them in every other respect, I am not able to say whether the local use of the sulphite was entitled to any credit.

